# Biomechanical effects of different staging and attachment designs in maxillary molar distalization with clear aligner: a finite element study

**DOI:** 10.1186/s40510-023-00493-0

**Published:** 2023-12-04

**Authors:** Jie Gao, Donghui Guo, Xu Zhang, Yuxun Cheng, Hao Zhang, Yuerong Xu, Zuolin Jin, Yanning Ma

**Affiliations:** 1 State Key Laboratory of Oral & Maxillofacial Reconstruction and Regeneration, National Clinical Research Center for Oral Diseases, Shaanxi Clinical Research Center for Oral Diseases, Department of Orthodontics, School of Stomatology, The Fourth Military Medical University, No. 169 Changle West Road, Xi’an, 710032 China; 2 Shanxi Medical University School and Hospital of Stomatology, Taiyuan, 030001 China

**Keywords:** Clear aligner, Molar distalization, Different staging design, Different attachment

## Abstract

**Background:**

In the present study, the effects of distalizations of one and two molars with different step distances and attachment designs have been analyzed.

**Methods:**

A 3D finite element analysis model has been developed in order to determine the tendency of tooth displacement and stress distribution with clear aligner treatment.

**Results:**

Under the condition of single-molar distalization, when the step distance was set to 0.25 mm, the total displacement was 0.086 mm for central incisors, 0.080 mm for lateral incisors, 0.084 mm for canines, 0.102 mm for the first premolar and 0.076 mm for the second premolar. The von Mises stress of roots and the principal stress of the periodontal ligament was slightly lower than in the control group when the step distance was set to 0.130 mm. Under the condition of two-molar distalization, when the step distance was set to 0.130 mm, the total displacements for central incisors, lateral incisors and canines as well as both the first and second maxillary molars were basically the same as with a distance of 0.250 mm for one-molar distalization. In addition, when the step distance was 0.130 mm with two-molar distalization, the rotation center of the first and second molar was closer to the apex of the root indicating that the smaller step distance led to more bodily movement during the two-molar distalization. However, displacement tendencies of the first molar and the second molar were basically the same whether horizontal or vertical rectangular attachments were added.

**Conclusions:**

A step distance of moving two molars to 0.130 mm can achieve the same reaction force on the anterior teeth as moving one molar 0.250 mm without effects on horizontal or vertical rectangular attachments.

**Clinical relevance:**

Our results provide a theoretical basis and guidance for simultaneously moving two molars backward in clinical practice using a clear aligner.

## Introduction

Clear aligner treatment (CAT) has become an attractive alternative for orthodontic therapy due to its efficient, esthetic and comfortable features. CAT is a personalized and custom-made thermoplastic product generated using computer-aided design and simulation from an initial impression or oral scan [1]. Clear aligners are initially applied for mild-to-moderate malocclusions, including slight crowding and lower incisor extractions [2–4]. Recent studies reported that CAT has been successfully used in more complex cases such as premolar extractions, open bite and decompensation treatment before orthognathic surgery, due to the enhanced controllability of aligners [5–7]. Maxillary molar distalization is a strategy to acquire a 2–3-mm arch space to achieve a Class I relationship [8], and CAT has become a new option for this treatment [9]. Using the Invisalign CAT system [10], Simon et al. reported that the efficacy of molar distalization with prescribed movement of at least 1.5 mm was 88% [11], whereas Saif et al. [12] postulated a prescribed movement of 2.6 mm with the same Invisalign treatment for successful maxillary molar distalization, with efficacies of 75.5% for maxillary first and 72.2% for second molars (72.2%). Recently, CAT has expanded its clinical applications thanks to the development of material science and finite element analysis (FEA), which is suitable to calculate the forces generated within different tissues, such as alveolar bone, the periodontal ligament (PDL) and teeth, and has recently been used in biomechanics to analyze the external forces in residential structures to simulate tooth displacement patterns in orthodontics [13, 14].

To increase the efficiency of molar distalization in CAT, staging and attachment designs, such as shape and position, are considered as crucial parameters. The classic clinical staging of molar distalization is at a single-tooth level following a V-pattern, which is cost-intensive and time-consuming. The distant movement of the molars in different staging designs, such as distal moving of two molars simultaneously in a V-pattern style, has been found to address such an issue [15]. In addition, other studies point to various specific auxiliary elements that facilitate complex tooth movements [16, 17].

Here, the aim was to determine the trends and stress distributions of both anterior and posterior teeth under different conditions of molar distalization designs and to observe the effect of different attachment designs on molar distalization with clear aligners. Using 3D FEA, we investigated the effects of single and combined first and second molar distalizations on the anterior teeth. The results will be a potential reference source for clinical application.

## Methods

**Fig. 1:**
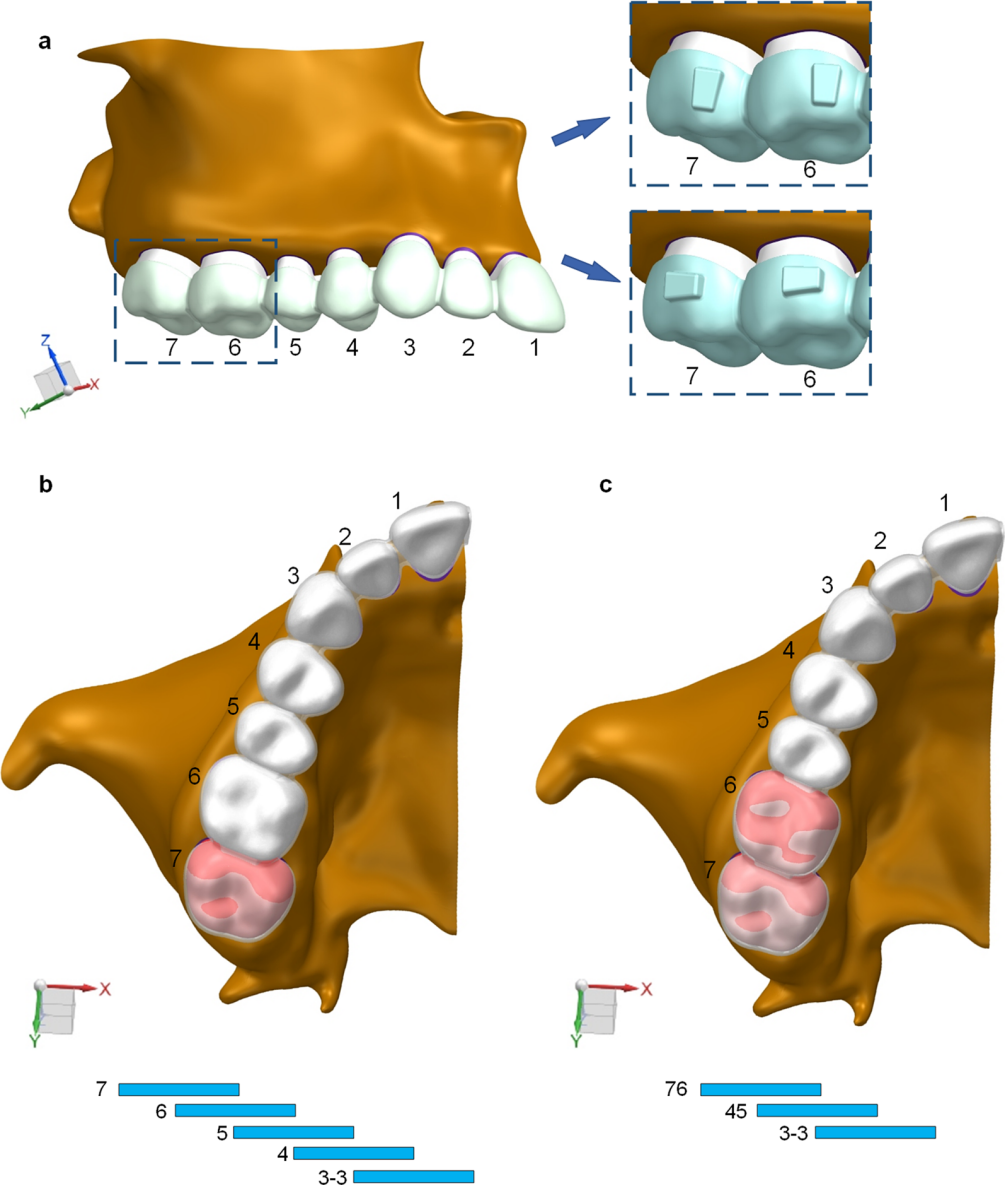
Three-dimensional FEA model for molar distalization. **a**, Maxillary arch with crowns, different buccal attachments (blue squares) and a geometric model of the clear aligner (X–Y-Z axes). **b**, one-molar distalization and “V-pattern” staging. **c**, two-molar distalization and “V-pattern” staging. 7: second molar 6: first molar 5: second premolar 4: first premolar 3–3: canine, lateral incisor, central incisor

A 3D finite element model was established. In particular, teeth and maxillary bone were reconstructed based on cone-beam computed tomography scanning of a healthy volunteer with well-aligned dentition and normal axial inclination of the upper incisors [14, 18]. Next, the data were imported into MIMICS 20.0 software (Materialise, Leuven, Belgium) to generate the 3D model. With the help of GEOMAGIC Studio 2014 (Raindrop GEOMAGIC, North Carolina, USA), the 3D model of maxillary bone and dentition was constructed. Finally, all components were imported into ANSYS Workbench 2019 (ANSYS, Pennsylvania, USA) to generate a 3D finite element model for FEA (Fig. 1).

### Establishment of a coordinate system

The X-axis represents the direction of the coronal plane with the positive direction being toward the mesial surface of the tooth. The Y-axis represents the sagittal plane with the positive direction being toward the lingual surface, and the Z-axis represents the vertical plane with the positive direction being toward the gingival tissue.

### Material properties

A rigid union condition without relative displacement (bonded) was established for the following interfaces: ligament-bone; tooth-ligament; and teeth-attachments. A no separation condition was constructed among teeth interfaces. All structures were assumed to be linear elastic isotropic and homogeneous materials. Their mechanical properties were obtained from previous studies and are shown in Table 1 [14, 18, 19]. Surface-to-surface contact was used between the aligner surface and teeth and attachments surfaces with a Coulomb friction coefficient of *μ* = 0.2.

**Table 1:** Properties of the materials considered in this study

Material	Young’s modulus, MPa	Poisson’s ratio
Alveolar bone	1.37 × 10^3^	0.26
Tooth	1.96 × 10^4^	0.3
PDL	6.9 × 10^–1^	0.49
Aligner	816	0.36
Attachment	12.5 × 10^3^	0.36

*PDL* Periodontal ligament

### Loading method

In the 3D model, the added forces in clinical usage were imitated. For this simulation, the second molar was first moved by 0.250 mm into the right distal position and the forces resulted in deformation of the aligner. The forces of the aligner deformation on each tooth were then calculated (ANSYS Workbench 2019) and loaded back onto the corresponding tooth in the reverse direction.

### Experimental design

According to the Invisalign recommendation, we set the initial tooth movements at 0.250 mm. For single maxillary molar distalization, 0.250 mm was set, while for combined maxillary molar distalization, 0.250 mm was set as the control, and the step distance was gradually decreased from 0.250 mm to 0.200 mm, 0.150 mm, 0.140 mm and 0.130 mm, respectively. In order to exclude the interference of attachments with the anterior and posterior teeth during the distal movement of the teeth, no attachments were designed for this part of the study. The stress distribution and displacement tendency of incisors, canine and premolars were analyzed and compared with the control group.

In this study, we designed the horizontal and vertical rectangular attachments according to the attachment size of the most widely used clear aligner in international, Invisalign, and other clear aligners comprised of the following dimensions: length × width × thickness: 3 × 2 × 1 mm.

The placement was in the mesial third of the tooth. Thus, four groups of attachments models were proposed: (1) single-molar distalization: horizontal rectangular attachments were designed for the first and second molars, the step distance was set to 0.250 mm; (2) vertical rectangular attachments were designed for the first and second molars, the step distance was set to 0.250 mm. For the two-molar distalization, the best step distance was chosen; (3) for two-molar distalization, the horizontal rectangular attachments were designed for the first and second molars, the step distance being set to 0.130 mm; and (4) vertical rectangular attachments were designed for the first and second molars, the step distance being set to 0.130 mm.

## Results

### The suitable step distance for the double-molar distalization mode

To test whether distalization of two molars at the same time was a better way than only distalization of one molar, we simulated both alternatives using 3D FEA.

**Fig. 2:**
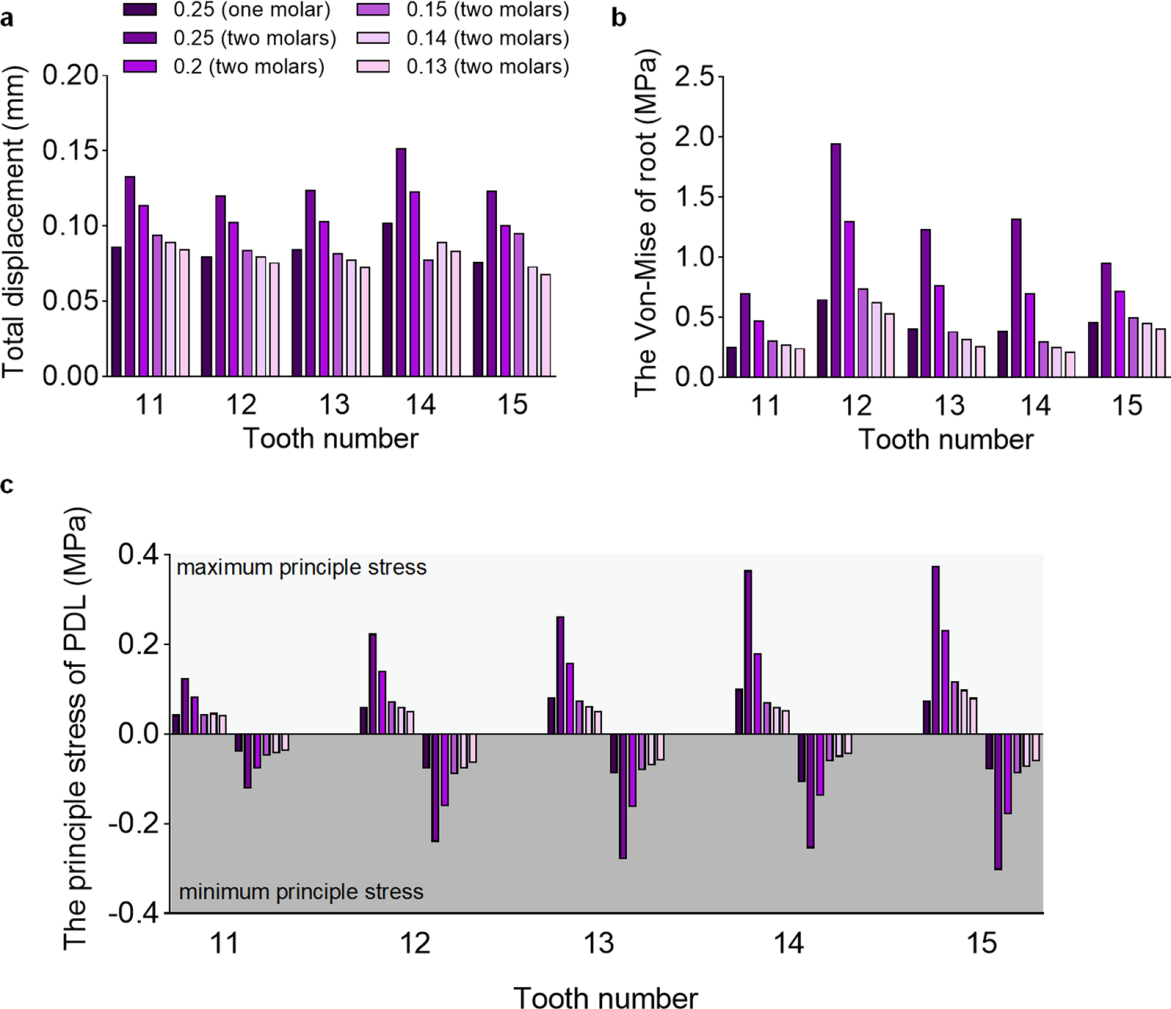
**a**, Total displacement of incisors, canine and premolars under the condition of different step distances. **b**, The root von Mises stress of incisors, canine and premolars. c, The PDL principle stresses of incisors, canine and premolars

As shown in Fig. 2, under the condition of single-molar distalization, when the step distance was set to 0.250 mm, the total displacement was 0.086 mm for central incisors, 0.080 mm for lateral incisors, 0.084 mm for canines, 0.102 mm for the first premolar and 0.076 mm for the second premolar. As for the magnitude of stress for different structures, the root of lateral incisors exhibited the highest von Mises stress (0.645 MPa) among the roots of teeth, while the von Mises stress of PDL increased from the anterior teeth to the posterior teeth (Fig. 2).

**Fig. 3:**
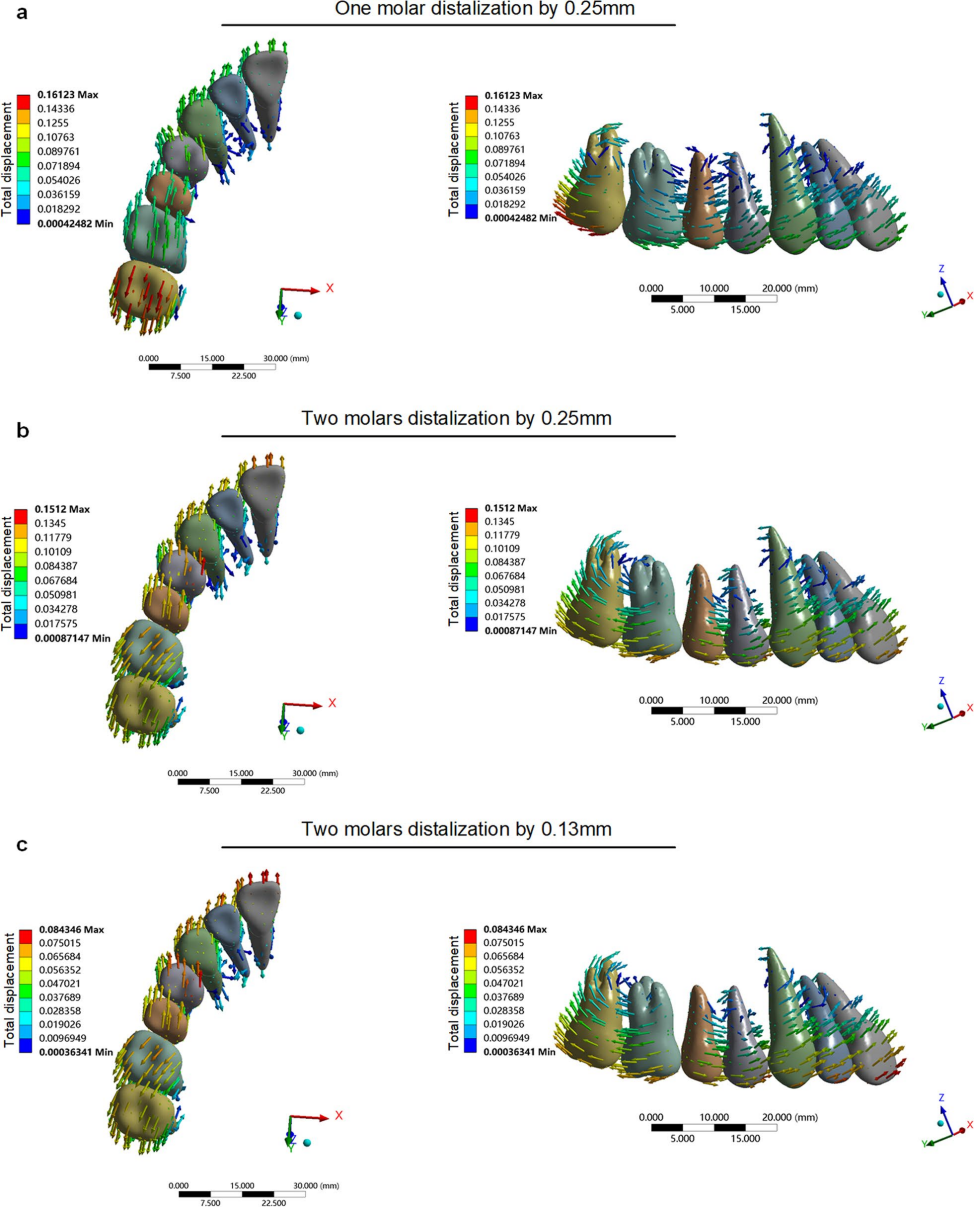
Total displacement of one-molar distalization with a step distance of 0.250 mm (**a**), two-molar distalization by 0.250 mm (**b**), and 0.130 mm (**c**), respectively. Arrows indicate the direction of tooth movement. The colder the tone, the less the tendency for tooth movement; the warmer the tone, the more the tendency for tooth movement

**Fig. 4:**
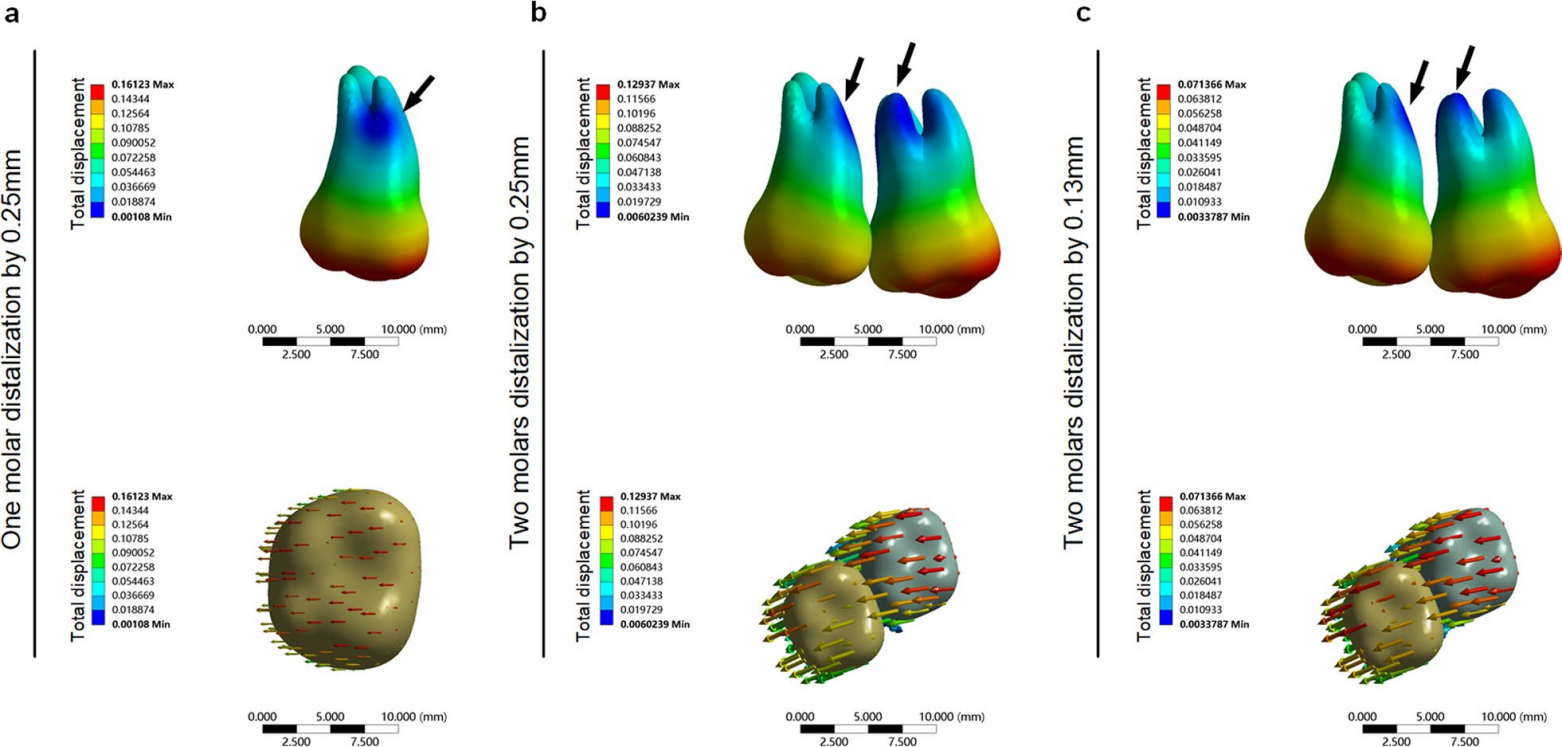
**a**, Second molar rotation of one-molar distalization by 0.250 mm. **b**, The first and second molar rotation of two-molar distalization by 0.250 mm. **c**, The first and second molar rotation of two-molar distalization by 0.130 mm. The blue part pointed at by the black arrow is the center of rotation

Under the condition of two-molar distalization, when the step distance was set to 0.250 mm, the total displacement was 0.133 mm for central incisors, 0.120 mm for lateral incisors, 0.123 mm for canines, 0.151 mm for the first premolar and 0.123 mm for the second premolar. In this experiment group, the total displacement for teeth was approximately 1.5 times that of the control group (Fig. 3). These results indicated that at a set step distance of 0.250 mm, moving 2 molars distal simultaneously on the anterior teeth was subject to a more significant reciprocal reaction force than distalization of only one molar alone. In addition, we observed that the center of rotation of the two-molar distalization was closer to the apex of the root than one-molar distalization at a set step distance of 0.250 mm, suggesting that moving two-molar distalization at the same time is more suitable for bodily movement (Fig. 4).

In addition, the total displacement of the anterior region decreased due to the step distance setup. Strikingly, when the step distance was set to 0.130 mm, the total displacement was slightly lower than in the control group. These results suggested that the anchorage consumption of the anterior teeth was the same when two-molar distalization with a step distance of 0.130 mm and one-molar distalization with a step distance of 0.250 mm. It was also observed that when the step distance was 0.130 mm, the rotation center of the first and second molar was closer to the apex of the root (Fig. 4), indicating that the smaller the step distance, the more bodily movement of two-molar distalization was visible. When the distance was set to 0.250 mm, as for the magnitude of stress for different structures, the von Mises stress of the roots and the principle stress of PDL were significantly higher than that of the control group (Fig. 2). Interestingly, the magnitude of stress decreased with the decrease in step distance. Figure 2 shows that the von Mises stress of roots and the principle stress of PDL was slightly lower than that of the control group when the step distance was set as 0.130 mm.

### Roles of different attachment designs in distal moving of the molar

#### Influence of different attachments setting on the teeth displacement tendency, stress value and the distribution of roots under the condition of single-molar distalization

**Fig. 5:**
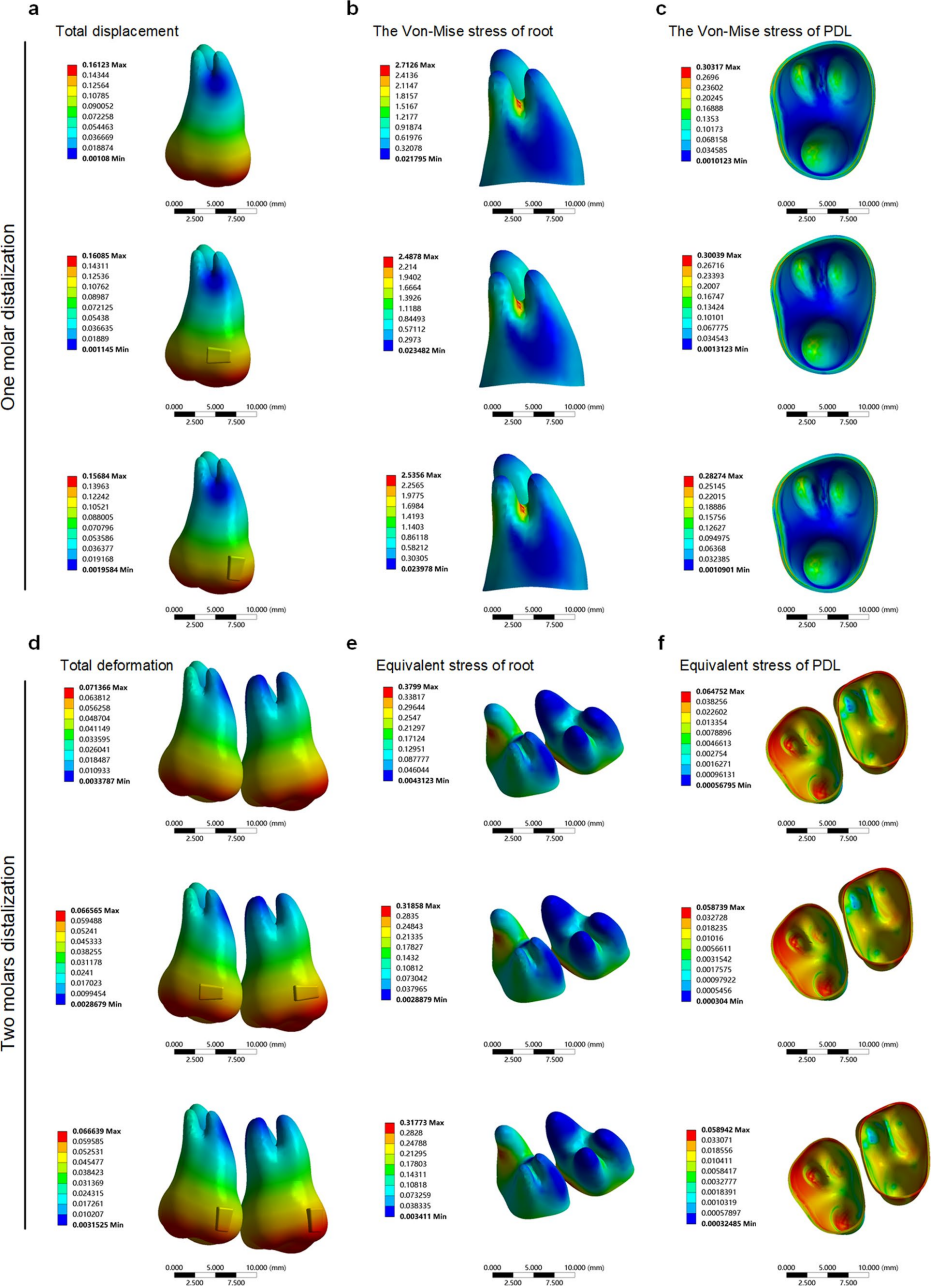
Total displacement, von Mises stress of the root and the von Mises stress of PDL of the molar with different attachment designs. **a**, One-molar distalization without an attachment. **b**, One-molar distalization with a horizontal attachment. **c**, One-molar distalization with a vertical attachment. **d**, Two-molar distalization without attachments. **e**, Two-molar distalization with horizontal attachments. **f**, Two-molar distalization with vertical attachments

The displacement tendency of the second molar was consistent with the apical 1/3 as the center of rotation, independent of the addition of the attachment in the control group. The instantaneous displacement value of the second molar was 0.161 mm without the addition of the attachment. The instantaneous displacement value of the second molar was 0.161 mm with the addition of the horizontal rectangular attachment. Furthermore, the instantaneous displacement value of the second molar was slightly decreased (0.157 mm) with the addition of the vertical rectangular attachment (Fig. 5), suggesting that attachments may play a minimal role in molar distalization.

Similarly, the displacement tendency of the anterior teeth in the three groups was basically consistent, but the instantaneous displacement value of the group without attachments was the largest. Taking the central incisors as an example, the instantaneous displacement value of the central incisors in the group without attachments was 0.086 mm, the instantaneous displacement value of the central incisors in the group with horizontal attachment was 0.080 mm, and the instantaneous displacement of the central incisors in the group with vertical attachment was 0.077 mm. In particular, the anterior teeth of the three groups showed an intrusion tendency in the vertical direction, especially the central incisors and the first premolars. Taking the central incisors as an example, the vertical instantaneous displacement value of the incisors in the group without attachments was 0.058 mm, the vertical instantaneous displacement of the incisors in the group with a horizontal attachment was 0.054 mm, and the vertical instantaneous displacement of the incisors in the group with a vertical attachment was 0.055 mm.

We analyzed the von Mises and the main stress of the anterior teeth roots to determine the influence of the attachments. The stress distribution for the three groups was basically consistent, and the von Mises and the maximum main stresses of the lingual and distal sides of lateral incisors were larger, indicating that this area was a stress concentration area. The stress value of the non-attachment group was greater than that of the horizontal and vertical attachment groups.

#### Influence of different attachments settings on the teeth displacement tendency and stress value and the distribution of roots under the condition of double-molar distalization

The displacement tendency of the first molar and second molar was basically the same whether the attachment existed or not. The displacement trend of the group without an attachment was the largest. Taking the first molar as an example, the instantaneous displacement value of the first molar without an attachment was 0.076 mm, the instantaneous displacement value of the first molar with a horizontal attachment was 0.067 mm, and the instantaneous displacement of the first molar in the group with a vertical rectangular attachment was 0.666 mm, indicating that the displacement tendency of the first molar without an attachment was slightly greater than that with an attachment.

The displacement trend of the anterior teeth in the three groups was basically the same, but the instantaneous displacement of the group without an attachment was the greatest. In particular, the anterior teeth of the three groups showed an intrusion tendency in the vertical direction, especially the central incisors. The instantaneous displacement of the central incisor in the group without an attachment was 0.019 mm, the instantaneous displacement of the central incisor in the group with a horizontal attachment was 0.016 mm, and the instantaneous displacement of the central incisor in the group with a vertical attachment was 0.016 mm. These results indicated that the anterior teeth were not only labially inclined, but also somewhat intruded by the reaction force (Table 2).

**Table 2:** Displacement tendency of the anterior teeth in the three groups

The displacement tendency (mm)		11	12	13
Without attachment	Total	0.089	0.080	0.077
	X	0.016	0.045	0.050
	Y	0.028	0.025	0.022
	Z	0.019	0.016	0.022
With horizontal attachment	Total	0.075	0.070	0.069
	X	0.013	0.042	0.045
	Y	0.023	0.021	0.019
	Z	0.016	0.015	0.020
With vertical attachment	Total	0.075	0.071	0.069
	X	0.013	0.042	0.046
	Y	0.023	0.021	0.019
	Z	0.016	0.015	0.020

11, the central incisor; 12, the lateral incisor; 13, the canine

The stress distribution for the three groups was basically consistent, and the von Mises and maximum main stresses of the lingual and distal sides of the lateral incisors were greater, indicating that this area was a stress concentration area. The stress value of the non-attachment group was greater than that of the horizontal and vertical attachment groups (Table 3). These results indicated that teeth without attachments move more smoothly during distal molar movement, possibly because the friction between the aligners and the teeth was increased by the attachments. The concentration of reaction forces on the anterior teeth was also focused in the lateral incisal area, suggesting that the lateral incisal area is a problematic area for CAT, a finding consistent with what has been generally observed in clinical practice.

**Table 3:** The von Mises stress of the anterior teeth and premolars root in the three groups

The von Mises stress of root (MPa)	11	12	13	14	15
Without attachment	0.268	0.627	0.316	0.248	0.449
With horizontal attachment	0.198	0.469	0.216	0.181	0.358
With vertical attachment	0.199	0.468	0.213	0.179	0.356

11, the central incisor; 12, the lateral incisor; 13, the canine; 14, the first premolar; 15, the second premolar

#### Influence of different attachment settings on the stress value and distribution of PDL and alveolar bone

The stress distribution and value of alveolar bone and PDL were measured and analyzed to evaluate the influence of different molar distalization modes and different attachment settings on the periodontal tissue. The results showed that the stress of the group with attachments was smaller than the group without attachments, whether with a single-molar distalization mode or a double-molar distalization mode. In particular, this trend was more obvious in the double-molar distalization mode.

## Discussion

The results of the study showed that when two molars were moved distal by 0.250 mm at the same time, the total displacement, the von Mises stress of the root and the main stress of PDL on the anterior regions were significantly greater compared to one-molar distalization. With a decrease in step distance, the stress decreased significantly. When the two molars moved posteriorly by 0.130 mm, the total displacement, the von Mises stress of the root and the principal stress of PDL on the anterior teeth were significantly reduced compared with one-molar distalization (Fig. 2). This study also found that the von Mises and maximum principal stress of the lingual side and the distal middle side of the lateral incisors were greater, indicating that this area was a stress concentration area. Moreover, it became clear that that distalization of two molars at the same time resulted in more bodily movement than distalization of one molar. The smaller the movement step, the more the center of rotation was shifted toward the apex of the root.

Saif et al. [12] used a superimposition model based on palatal rugae registration to investigate maxillary molar distalization efficiency. They found that the most teeth affected by anchorage loss during molar distalization movement were the central incisors (39.9%), followed by the lateral incisors (37.4%), but the canines (22.7%) were less affected. From previous observations, the anterior teeth anchorage can be strengthened using auxiliary devices such as Class II elastics and temporary anchorage devices to prevent side effects during molar distalization [20–23].

Different attachment designs may affect tooth movement. Ravera et al. [24] found that the first molars moved more distally with an attachment and that there was no significant difference in the amount of second molar movement with or without vertical rectangular attachments. Dai et al. [6, 25] found that optimized and horizontal rectangular attachments had better mesiodistal control than vertical rectangular attachments. In the present study, only conventional rectangular (horizontal and vertical) attachments were used. For clinical bonding habits and convenience, the attachment was placed in the mesial third of the molar, slightly near the incisal of the tooth. We found no difference in the effects of horizontal and vertical rectangular attachments on the strain stress and displacement tendency of molar distalization. In addition, the use of attachments had no significant effect on the outcome of molar distalization movement, a finding in good agreement with previous studies [12, 16, 24]. Using FEA, Ayidağa et al. [26] found that a vertical rectangular attachment in a clear aligner reduced the mesiodistal tipping tendency during molar distalization. However, they only used a FEA for the first maxillary molar, which was different from our model and loading style.

The present research differs from other studies because when the molar moved distally, a clear aligner stuck into the contact of the teeth, and the aligner became longer. Therefore, the attachments may play a relatively minor role in distalization of molars. However, after the molars are moved into position, the posterior teeth need better control when the anterior teeth move. Then, the attachments may play a more critical role.

Limitations of the present study are methodical problems inherited in the FEA analysis regarding high dependency on mesh, the number of elements into which the region is divided, as well as their shape and arrangement [27]. In addition, since bone, PDL and teeth are complex non-homogenous structures that are simplified in FEA to be adapted for calculations, the FEA model may not be conclusive and must be supported with further clinical research.

## Conclusions

Based on a FEA model for maxillary molar distalization with clear aligners, the reciprocal reaction force on the anterior region for distalization of two molars was greater than with one-molar distalization for the same step distance. Reducing the step distance of moving two molars to 0.130 mm can achieve the same reaction force on the anterior teeth as moving one molar 0.250 mm. In addition, distalization of two molars at the same time is more effective for bodily teeth movements than distalization of only one molar. The FEA model also showed that the use of horizontal and vertical attachments had only minor effects on the maxillary molar distalization movement.

## Data Availability

The datasets used and/or analyzed during the current study are available from the corresponding author on reasonable request.

## Abbreviations

CAT: Clear aligner treatment
FEA: Finite element analysis
PDL: Periodontal ligament

## References

[1] Weir T (2017). Clear aligners in orthodontic treatment. Aust Dent J.

[2] Lagravère MO, Flores-Mir C (2005). The treatment effects of Invisalign orthodontic aligners: a systematic review. J Am Dent Assoc.

[3] Kravitz ND, Kusnoto B, BeGole E, Obrez A, Agran B (2009). How well does Invisalign work? A prospective clinical study evaluating the efficacy of tooth movement with Invisalign. Am J Orthod Dentofac Orthop.

[4] Zawawi KH (2014). Orthodontic treatment of a mandibular incisor extraction case with Invisalign. Case Rep Dent.

[5] Caruso S, Nota A, Ehsani S, Maddalone E, Ojima K, Tecco S (2019). Impact of molar teeth distalization with clear aligners on occlusal vertical dimension: a retrospective study. BMC Oral Health.

[6] Dai FF, Xu TM, Shu G (2021). Comparison of achieved and predicted crown movement in adults after 4 first premolar extraction treatment with Invisalign. Am J Orthod Dentofac Orthop.

[7] Cong A, Ruellas ACO, Tai SK, Loh CT, Barkley M, Yatabe M (2022). Presurgical orthodontic decompensation with clear aligners. Am J Orthod Dentofac Orthop.

[8] Bolla E, Muratore F, Carano A, Bowman SJ (2002). Evaluation of maxillary molar distalization with the distal jet: a comparison with other contemporary methods. Angle Orthod.

[9] D'Antò V, Valletta R, Ferretti R, Bucci R, Kirlis R, Rongo R (2023). Predictability of maxillary molar distalization and derotation with clear aligners: a prospective study. Int J Environ Res Public Health.

[10] Align T (2002). The Invisalign reference guide.

[11] Simon M, Keilig L, Schwarze J, Jung BA, Bourauel C (2014). Forces and moments generated by removable thermoplastic aligners: incisor torque, premolar derotation, and molar distalization. Am J Orthod Dentofac Orthop.

[12] Saif BS, Pan F, Mou Q, Han M, Bu W, Zhao J (2022). Efficiency evaluation of maxillary molar distalization using Invisalign based on palatal rugae registration. Am J Orthod Dentofac Orthop.

[13] Jiang T, Wu RY, Wang JK, Wang HH, Tang GH (2020). Clear aligners for maxillary anterior en masse retraction: a 3D finite element study. Sci Rep.

[14] Cheng Y, Gao J, Fang S, Wang W, Ma Y, Jin Z (2022). Torque movement of the upper anterior teeth using a clear aligner in cases of extraction: a finite element study. Prog Orthod.

[15] Bondemark L, Kurol J (1992). Distalization of maxillary first and second molars simultaneously with repelling magnets. Eur J Orthod.

[16] Gomez JP, Peña FM, Martínez V, Giraldo DC, Cardona CI (2015). Initial force systems during bodily tooth movement with plastic aligners and composite attachments: a three-dimensional finite element analysis. Angle Orthod.

[17] Barone S, Paoli A, Razionale AV, Savignano R (2017). Computational design and engineering of polymeric orthodontic aligners. Int J Numer Method Biomed Eng.

[18] Ma Y, Li S (2021). The optimal orthodontic displacement of clear aligner for mild, moderate and severe periodontal conditions: an in vitro study in a periodontally compromised individual using the finite element model. BMC Oral Health.

[19] Ahmed T, Padmanabhan S, Pottipalli SH (2023). Effects of varying attachment positions on palatal displacement of maxillary incisors with clear aligner therapy: a three-dimensional finite element analysis. J Orofac Orthop.

[20] Patterson BD, Foley PF, Ueno H, Mason SA, Schneider PP, Kim KB (2021). Class II malocclusion correction with Invisalign: is it possible?. Am J Orthod Dentofac Orthop.

[21] Kinzinger GS, Fritz UB, Sander FG, Diedrich PR (2004). Efficiency of a pendulum appliance for molar distalization related to second and third molar eruption stage. Am J Orthod Dentofac Orthop.

[22] Acar AG, Gürsoy S, Dinçer M (2010). Molar distalization with a pendulum appliance K-loop combination. Eur J Orthod.

[23] Auladell A, De La Iglesia F, Quevedo O, Walter A, Puigdollers A (2022). The efficiency of molar distalization using clear aligners and mini-implants: Two clinical cases. Int Orthod.

[24] Ravera S, Castroflorio T, Garino F, Daher S, Cugliari G, Deregibus A (2016). Maxillary molar distalization with aligners in adult patients: a multicenter retrospective study. Prog Orthod.

[25] Dai FF, Xu TM, Shu G (2019). Comparison of achieved and predicted tooth movement of maxillary first molars and central incisors: first premolar extraction treatment with Invisalign. Angle Orthod.

[26] Ayidağa C, Kamiloğlu B (2021). Effects of variable composite attachment shapes in controlling upper molar distalization with aligners: a nonlinear finite element study. J Healthc Eng.

[27] Chopade S, Madhav V, Palaskar J (2014). Finite element analysis: new dimension in prosthodontic research. J Dent Allied Sci.

